# Hormonal, metabolic and inflammatory circulating biomarker profiles in obese and non-obese Brazilian middle-aged women

**DOI:** 10.1371/journal.pone.0222239

**Published:** 2019-09-11

**Authors:** Leonardo Victor Galvão-Moreira, Anna Cyntia Brandão Nascimento, Izabella Mikaella Souza Campos D'Albuquerque, Marcus Antonio Silva Sousa, Haissa Oliveira Brito, Maria do Desterro Soares Brandão Nascimento, Maria Bethânia da Costa Chein, Luciane Maria Oliveira Brito

**Affiliations:** 1 School of Medicine, Federal University of Maranhão, São Luís, Brazil; 2 Postgraduate Program in Adult Health, Federal University of Maranhão, São Luís, Brazil; University of South Alabama Mitchell Cancer Institute, UNITED STATES

## Abstract

**Aim:**

To investigate circulating hormonal, metabolic and inflammatory biomarker profiles in obese and non-obese middle-aged women.

**Methods:**

A total of 110 women, aged 40–60 years, were included in this cross-sectional study. Patients were allocated, according to the occurrence of menopause and body mass index (BMI), into four groups: PM0 (premenopausal non-obese), PM1 (premenopausal obese), M0 (postmenopausal non-obese), and M1 (postmenopausal obese). Serum levels of gonadotropins, sex hormones, lipid markers, leptin, hs-CRP and interleukin-6 were obtained using either colorimetric or immunoenzymatic assays. Univariate and correlation analyses were performed among all clinical and laboratorial parameters. Principal component analysis was used to characterize subsets of biomarkers, which had their discriminatory capacity tested using discriminant function analysis.

**Results:**

Levels of gonadotropins and female sex hormones were similar between PM0 and PM1 and between M0 and M1 (p > 0.05), all of them varied between PM0 and M0 (p < 0.05), but only estradiol was significantly altered in the comparison between PM1 and M1 (p = 0.027). Regarding metabolic markers, leptin was lower in PM0 than in M0 (p = 0.010) and higher in M1 than in M0 (p = 0.046). In premenopausal women, BMI correlated only to leptin, while it correlated to several other markers in postmenopausal women. A combination of FSH and leptin serum levels significantly discriminated the four groups (Wilks’s lambda < 0.001, in canonical functions 1 and 2).

**Conclusion:**

A combined analysis of hormonal biomarkers may potentially distinguish obese from non-obese women with distinct menopause status. Further research is thus required to clarify the clinical significance of such findings.

## 1. Introduction

Menopause consists in the spontaneous or iatrogenic definitive cessation of menstrual cycles that follows the declining of ovarian follicular activity [1]. Its occurrence has been associated with a biological acceleration of aging and increased risk of death by cardiovascular diseases (CVD) and cancers [2–3]. Obesity is a well-known risk factor for both CVD and cancer, being also more prevalent in postmenopausal women [4–6]. In this context, several biomarkers of metabolic risk have been evaluated in postmenopausal women, attempting to explain the epidemiologic connection between menopause and CVD [7]; however, laboratory findings observed remain widely controversial.

Interestingly, subclinical metabolic dysfunctions may occur even in postmenopausal women who are not overweight or obese. Conversely, there is a group of overweight or obese individuals that are metabolically healthy [8]. Therefore, it has been increasingly hypothesized that currently available biomarkers may eventually fail to identify subclinical metabolic disorders. Indeed, hormones involved in the regulation of food intake such as leptin generally have a high sensitivity for discriminating obese from non-obese women. Nevertheless, pre- and postmenopausal women have presented with similar levels of leptin [9]. Leptin was also not associated with incident CVD events in postmenopausal patients [10].

Additionally, even though the use of lipid ratios for CVD risk assessment has been widely recommended [11], a recent study showed them to be unable to differentiate subclinical atherosclerosis between pre- and postmenopausal women [12]. Yet, several inflammatory biomarkers have been associated with coronary heart disease in midlife women, especially those with obesity [13]. Hence, considering that obese middle-aged women are at increased risk of death by several diseases, the present study was aimed at investigating the discriminatory potential of a combination of circulating biomarkers in obese and non-obese women with different menopause status.

## 2. Methods

This cross-sectional study was approved by the Institutional Review Board of the University Hospital of the Federal University of Maranhão (HUUFMA), São Luís, Brazil (protocol #698.706). Each patient was required to sign an informed consent before entry into the study. The present investigation was conducted according to the principles of the Declaration of Helsinki.

### 2.1 Participant selection

The recruitment of patients was carried out at Gynecological Endocrinology outpatient clinics of HUUFMA. A total of 66 premenopausal (PM) and 44 postmenopausal (M) women, aged 40–60 years, were included. Definition of both pre- and postmenopausal phases were based on the Stages of Reproductive Aging Workshop (STRAW) criteria [14]. All of the study participants were judged to be in good health based on medical history and physical examination. The following exclusion criteria were applied: any hormonal or drug treatments in the prior three months; individuals with any endocrine, cardiovascular, hepatic, renal, rheumatic or chronic respiratory disease; history of any type of cancer in the past 5 years; women with class III obesity (BMI ≥ 40).

### 2.2 Determination of body mass index (BMI) and formation of groups

The BMI was obtained by dividing weight (kg) by the square of height (m) and classified, following the WHO criteria and cut-off points, in normal weight, overweight or obese [15]. The patients' weight was measured using a digital weight scale (Welmy^®^; capacity of 180 kg; precision of 100 g), while height was measured using a stadiometer (capacity of 210 cm; precision of 0.1 cm). Patients were then divided, according to the occurrence of menopause and BMI, into four groups: PM0 (premenopausal non-obese, n = 49), PM1 (premenopausal obese, n = 17), M0 (postmenopausal non-obese, n = 27), and M1 (postmenopausal obese, n = 17).

### 2.3 Blood collection and storage

Blood collection was performed in the morning shift with a 12-hour fasting period. Patients were instructed to avoid alcohol consumption for at least 72 hours prior to the collection of blood samples. From each patient, 20 mL of whole blood was collected and stored under sterile vacuum tubes containing EDTA (for blood count) and serology (separating gel) without anticoagulant for biochemical and hormonal evaluation. Blood samples processing was conducted at most 1 h after collection, and serum samples were stored at -80°C for the subsequent analyses [16].

### 2.4 Measurement of circulating hormonal, metabolic and inflammatory biomarkers

Serum hormones levels, lipid profile and fasting glucose were analyzed using enzymatic colorimetric methods. Follicle-stimulating hormone (FSH), luteinizing hormone (LH), estradiol, progesterone, total cholesterol (TC), high-density lipoprotein (HDL-c), triglycerides (TG) and high sensitivity C-reactive protein (hs-CRP) were measured using a COBAS 6000 automated analyzer (Roche Diagnostics, Mannheim–Germany), following the manufacture instructions. The concentrations of low-density (LDL-c) and very low-density lipoproteins (VLDL-c) were calculated through the Friedwald formula (VLDL = TG/ 5; LDL-c = TC–HDL-c–VLDL-c) for TG values of up to 4.5 mmol/L [16–17]. Circulating leptin and iterleukin(IL)-6 levels were estimated using enzyme-linked immunosorbent assay (Quantikine^®^ ELISA kits, R&D Systems, Inc., Minneapolis, MN, USA), according to the manufacture instructions. All experiments were conducted in duplicate.

### 2.5 Statistical analysis

Means, standard deviations and medians of parameters were calculated. The student t or Mann-Whitney tests, one-way analysis of variance (ANOVA) and Bonferroni multiple comparisons or Kruskal-Wallis with Dunn’s multiple comparisons and Bonferroni correction, when appropriate, were used to compare clinical and laboratory parameters among groups. Kendall’s tau b correlations were performed to evaluate associations between all variables. Missing data were not excluded from the analysis, as nonparametric approaches were applied. Principal component analysis (PCA) and discriminant function analysis were utilized as previously described [18]. Briefly, PCA was used as a data reduction technique, hereby reducing the number of variables, exploring their associations and selecting biomarkers with the greatest explanatory variance. Next, a discriminant function analysis was built based on the four principal components obtained to evaluate a combination of variables with predictive ability among the groups and subgroups. Statistical analyses were performed using SPSS 25.0 (SPSS Inc., Chicago, IL, USA).

## 3. Results

### 3.1 Comparison of clinical parameters and plasma biomarkers according to the menopause status

Table 1 illustrates the comparison of variables between premenopausal (PM) and postmenopausal (M) women. The group of premenopausal women was younger as compared to the group of postmenopausal participants (< 0.001). Conversely, the medians of BMI were similar between the two groups (p = 0.119). Gonadotropins were increased and sex hormones decreased in postmenopausal participants (p < 0.001). In the comparison of metabolic risk markers, only TC was significantly augmented in postmenopausal females (p = 0.008). Increased blood glucose and leptin values were also observed in postmenopausal as compared to premenopausal women (p = 0.05). The levels of other biomarkers analyzed were similar in both groups.

**Table 1:** Univariate comparison of clinical variables and serum biomarkers between pre- and postmenopausal women with and without obesity.

Variable	Premenopausal women			Postmenopausal women			p0	p1	p2	p3	p4	p5	p6	p7
	PM	PM0	PM1	M	M0	M1								
Age (year)^a^	43.0±5.45	43.07±5.33	42.66±6.21	54.45±6.92	55.03±7.43	54.53±7.62	<0.001	<0.001	>0.99	<0.001	<0.001	<0.001	<0.001	>0.99
BMI^b^	27.11	26.5	33.28	28.94	27.12	32.0	0.119	<0.001	<0.001	>0.99	<0.001	<0.001	>0.99	<0.001
FSH (mUI/dL)^b^	6.18	6.18	7.64	64.24	69.80	63.13	<0.001	<0.001	0.71	<0.001	0.004	0.003	0.128	0.44
LH (mUI/dL)^b^	6.75	6.06	7.90	29.44	29.84	26.56	<0.001	<0.001	0.58	<0.001	0.004	0.021	0.186	0.686
Estradiol (pg/dL)^b^	76.36	72.62	107.5	10.98	9.47	16.73	<0.001	<0.001	0.596	<0.001	0.010	<0.001	0.027	0.253
Progesterone (ng/dL)^b^	0.60	0.60	0.55	0.31	0.33	0.29	<0.001	0.003	0.949	0.013	0.058	0.243	0.297	0.813
TC (mg/dL)^a^	188.76±39.14	191.32±39.57	177.0±36.31	208.32±33.57	207.13±32.61	211.07±36.92	0.008	0.036	>0.99	0.382	0.526	0.116	0.144	>0.99
HLD-c (mg/dL)^a^	47.10±9.82	46.65±9.87	48.41±10.62	50.30±12.42	46.73±12.64	55.15±11.52	0.137	0.107	NS	NS	NS	NS	NS	NS
LDL-c (mg/dL)^a^	116.56±34.51	120.65±34.78	103.3±37.51	130.47±31.21	129.43±28.62	131.84±32.76	0.035	0.08	NS	NS	NS	NS	NS	NS
VLDL-c (mg/dL)^b^	22.0	22.0	22.0	22.0	24.50	22.0	0.327	0.669	NS	NS	NS	NS	NS	NS
TG (mg/dL)^a^	125.49± 73.11	127.65±78.80	127.58±61.23	137.69±72.48	151.30± 84.31	120.61±48.46	0.393	0.626	NS	NS	NS	NS	NS	NS
Glucose (mg/dL)^a^	91.43±11.24	90.91±10.37	91.25±6.49	95.39±9.18	94.50±9.69	99.38±7.18	0.056	0.097	NS	NS	NS	NS	NS	NS
Leptin (ng/mL)^b^	6.46	5.31	14.29	10.69	8.02	13.78	0.059	0.001	0.113	0.010	0.006	0.367	0.952	0.046
hs-CRP (mg/L)^b^	0.10	0.10	0.16	0.14	0.10	0.20	0.672	0.641	NS	NS	NS	NS	NS	NS
IL-6 (ng/mL)^b^	0.01	0.01	0.23	0.32	0.22	0.46	0.272	0.226	NS	NS	NS	NS	NS	NS

^a^ p values were obtained using the Student test or one-way ANOVA; the Bonferroni test was used for pairwise comparisons when appropriate.

^b^ p values were obtained using the Mann-Whitney or Krukal-Wallis test; the Dunn’s test was used for pairwise comparisons when appropriate; for p2-p6, Bonferroni correction was applied.

p0: PM x M; p1: PM0 x PM1 x M0 x M1; p2: PM0 x PM1; p3: PM0 x M0; p4: PM0 x M1; p5: PM1 x M0; p6: PM1 x M1; p7: M0 x M1.

NS: non-significant.

As illustrated in Table 2, age correlated to gonadotropins and sex hormones in premenopausal women; however, no correlation with age was found in the postmenopausal group. BMI positively correlated to leptin, while it correlated to several markers in postmenopausal women, including a negative correlation with FSH. Gonadotropins did not correlate to the majority of metabolic or inflammatory markers, except for TC, but this correlation was shown only in the PM group. Similarly, no correlation was found between sex hormones and metabolic/ inflammatory markers in both groups. Notably, no biomarker of lipidomic profile correlated to the BMI in the two groups. Fasting glucose correlated to the BMI and leptin only in postmenopausal women, and correlations among inflammatory markers were similar between the groups.

**Table 2:** Correlations observed among clinical variables and serum biomarkers in pre- and postmenopausal women.

Variable	Variables correlated (R coefficient)	
	Premenopausal women	Postmenopausal women
Age	FSH (0.418**); LH (0.356**); estradiol (-0.176*); progesterone (-0.293**).	None.
BMI	Leptin (0.592**).	FSH (-0.403**); fasting glucose (0.349*); leptin (0.326*); IL-6 (0.308*).
FSH	Age (0.418**); LH (0.593); estradiol (-0.498**); progesterone (-0.502**); total cholesterol (0.211*).	BMI (-0.403**); LH (0.623**); estradiol (-0.452**); progesterone (-0.249*)
LH	Age (0.356**); FSH (0.593**); estradiol (-0.257*); progesterone (-0.292**); total cholesterol (0.180*).	FSH (0.623**); estradiol (-0.265*).
Estradiol	Age (-0.176*); FSH (-0.498**); LH (-0.257*); progesterone (0.412**).	FSH (-0.452**); LH (-0.265*); progesterone (0.363**).
Progesterone	Age (-0.293**); FSH (-0.502**); LH (-0.292**); estradiol (0.412**).	FSH (-0.249*); estradiol (0.363**).
Total cholesterol	FSH (0.211*); LH (0.180*); LDL (0.792**); triglycerides (0.310**); VLDL (0.310**).	LDL (0.802**).
HDL	Fasting glucose (-0.192*); triglycerides (-0.322**); VLDL (-0.325**).	Triglycerides (-0.473**); VLDL (-0.472**).
LDL	Total cholesterol (0.792**); triglycerides (0.189*); VLDL (0.188*).	Total cholesterol (0.802**).
VLDL	Total cholesterol (0.310**); HDL (-0.325**); LDL (0.188*); fasting glucose (0.170*); triglycerides (0.988**)	HDL (-0.472**); fasting glucose (0.242*); triglycerides (0.988**); leptin (0.212*).
Triglycerides	Total cholesterol (0.310**); HDL (-0.322**); LDL (0.189*); VLDL (0.988**).	HDL (-0.473**); VLDL (0.988**); fasting glucose (0.242*).
Fasting glucose	HDL (-0.192*); VLDL (0.170*).	BMI (0.349*); leptin (0.277*); VLDL (0.242*); triglycerides (0.242*)
Leptin	BMI (0.592**); CRP (0.222*).	BMI (0.326*); VLDL (0.212*); fasting glucose (0.277*).
CRP	Leptin (0.222*); IL-6 (0.492**).	IL-6 (0.365**).
IL-6	CRP (0.492**).	BMI (0.308*); CRP (0.365**).

*p < 0.05,

**p < 0.01 for R coefficients obtained using Kendall’s tau b correlation.

### 3.2 Comparison of plasma biomarkers between obese and non-obese women with different menopause status

In Table1, when participants were clustered into four subgroups, taking into account both BMI and menopause status, the age and BMI did not vary (p > 0.05) within non-obese patients, either pre- or postmenopausal (PM0 and M0), as well as within obese participants (PM1 a M1). Levels of gonadotropins and sex hormones were similar between PM0 and PM1 and between M0 and M1 (p > 0.05). FSH, LH and progesterone levels significantly varied (p < 0.05) between non-obese women (PM0 and M0), but not between obese individuals (PM1 and M1, p > 0.05). Interestingly, only estradiol was significantly altered in the comparison between PM1 and M1 (p = 0.027).

**Fig 1:**
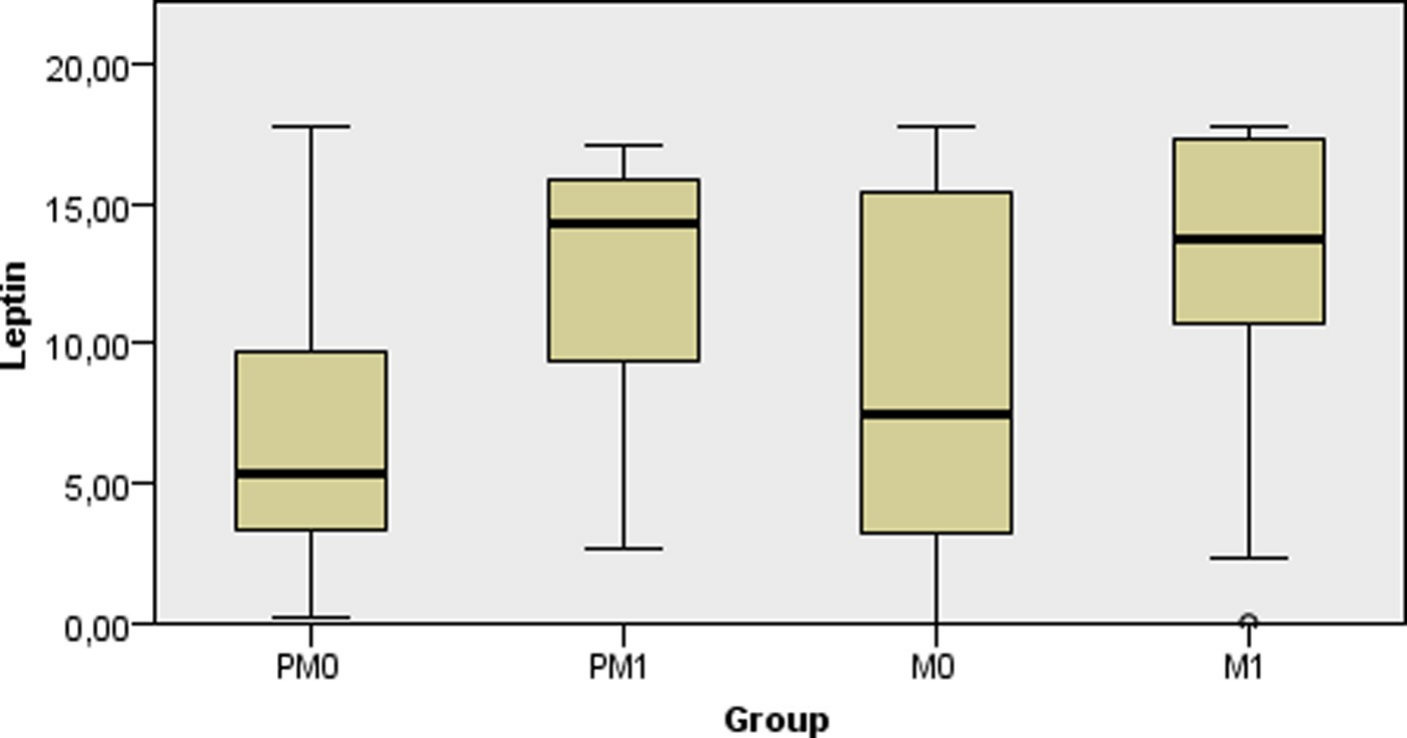
Comparison of medians of plasma leptin levels (ng/mL) between non-obese and obese women with different menopausal status. PM0: premenopausal non-obese; PMI: premenopausal obese; M0: postmenopausal non-obese; M1: postmenopausal obese.

**Fig 2:**
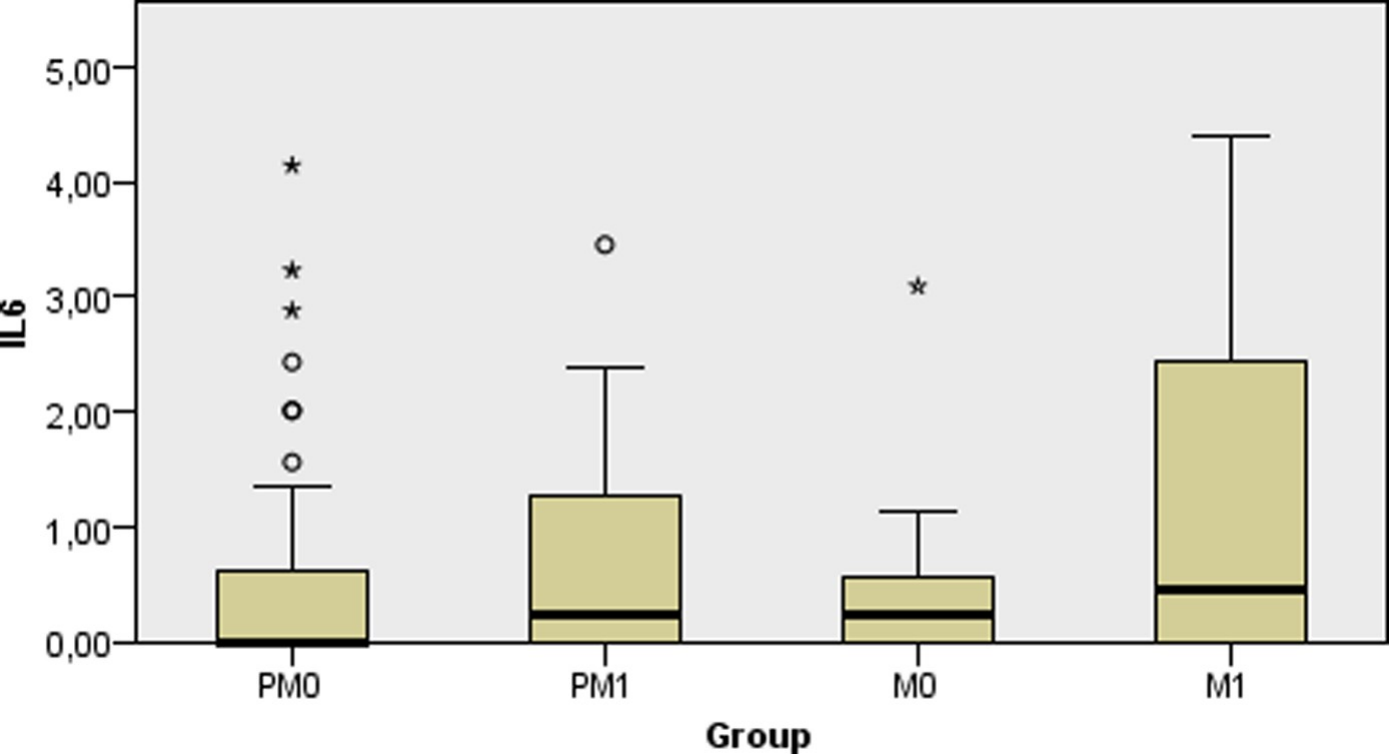
Comparison of medians of plasma IL-6 levels (ng/mL) between non-obese and obese women with different menopausal status. PM0: premenopausal non-obese; PMI: premenopausal obese; M0: postmenopausal non-obese; M1: postmenopausal obese.

Regarding the lipidomic profile, none of the biomarkers analyzed varied significantly among the four groups; fasting glucose and hs-CRP were also similar between the groups (Table 1, p > 0.05). As displayed in Fig 1, PM0 women presented with lower leptin levels as compared to M0 (p = 0.010), while higher leptin values were shown in the M1 group in comparison to PM0 (p = 0.006) and M0 (p = 0.046). Fig 2 shows that all groups had similar circulating levels of IL-6 (p > 0.05).

### 3.3 Discriminatory ability of plasma biomarker profiles

The number of components derived from PCA and the variation explained by these components in our sample are presented in Table 3. PCA revealed four principal components (PC) that had their discriminatory potential evaluated using discriminant function analysis. We tested whether each PC could distinguish premenopausal from postmenopausal women or discriminate individuals with distinct BMI scores. PC1 and PC4 were able to significantly discriminate pre from postmenopausal women (Wilks’s lambda < 0.05), while PC 2 and 4 discriminated BMI groups (Wilks’s lambda < 0.05); however, latent variables inferred relied mainly upon one single biomarker in all cases.

**Table 3:** Principal components (PC) and their respective percentages of variance derived from PCA are shown for the whole sample. Discriminatory potential of subsets of biomarkers derived from PCA was tested using discriminant function analysis for the comparison between pre- and postmenopausal women and according to the BMI classification.

	PC1	PC2	PC3	PC4
Biomarker (*)	FSH (0.877)	IL-6 (0.853)	HDL (0.851)	Fasting glucose (0.677)
	LH (0.852)	CRP (0.836)	Triglycerides (-0.775)	Leptin (0.651)
	Progesterone (-0.688)	Leptin (0.302)		LDL (0.463)
	Estradiol (-0.587)			
**Variance**	23.58%	15.34%	14.6%	10.34%
**Cumulative variance**	23.58%	38.93%	53.53%	63.87%
**Eigenvalue A**	0.458	0.055	0.062	0.102
**Eigenvalue B**	-	0.310	-	0.263
**Canonical correlation A**	0.560	0.228	0.242	0.304
**Canonical correlation B**	-	0.487	-	0.456
Wilks’s lambda A (**)	0.686 (<0.001)	0.948 (0.253)	0.941 (0.092)	0.908 (0.042)
Wilks’s lambda B (**)	-	0.763 (<0.001)	-	0.792 (<0.001)

*Biomarkers in each PC are displayed with factor loads (in parentheses), which indicate the correlation of individual markers on each component. Varimax rotation with Kaiser normalization was applied.

**Significance level.

A: variables were tested for the comparison between pre- and postmenopausal women; BMI was included as an independent variable in the model. B: variables were tested for the comparison according to the BMI classification (normal weight, overweight and obese); age was included as an independent variable in the model.

**Fig 3:**
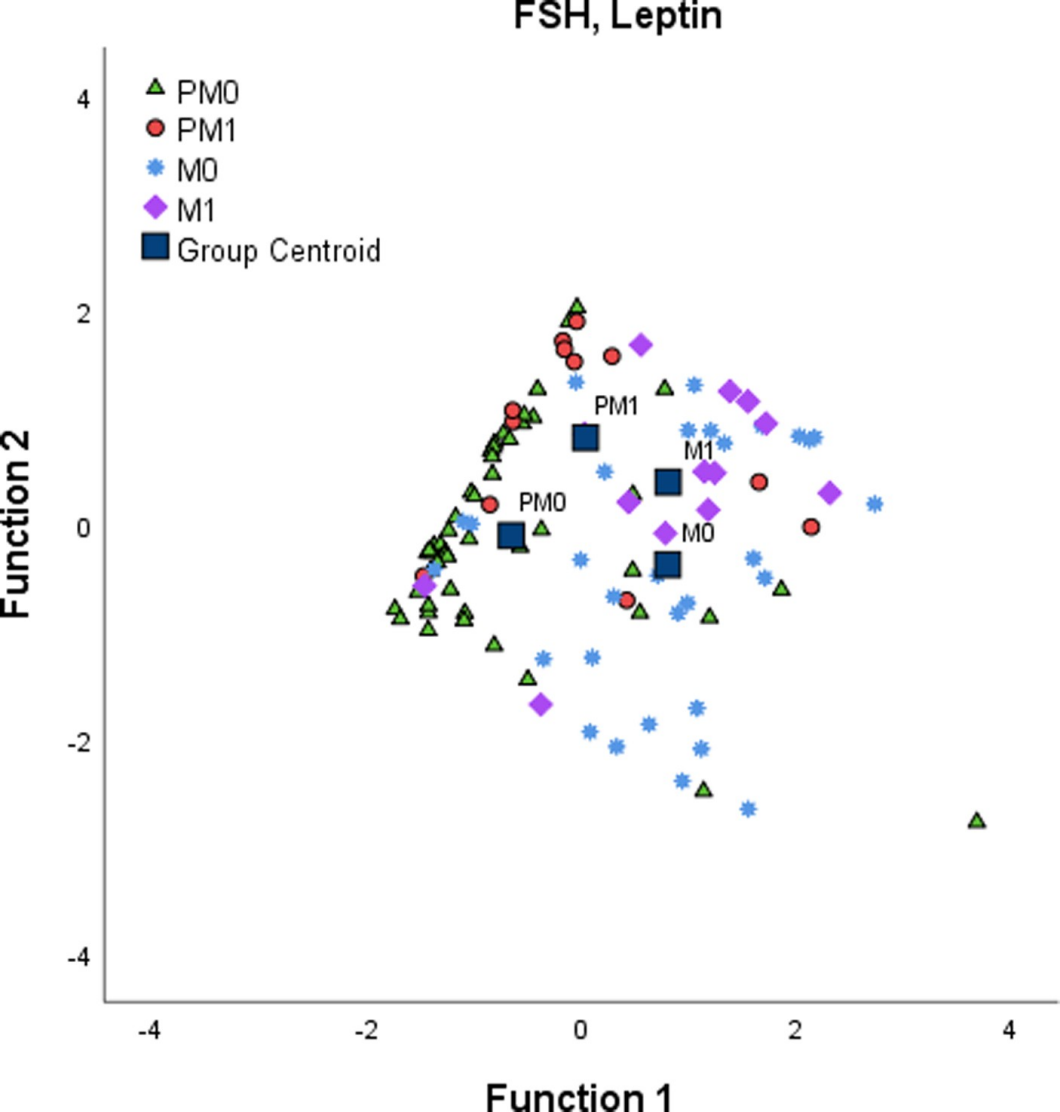
Discriminatory potential of serum biomarkers between the subgroups PM0, PM1, M0 and M1. The panel plot displays a significant separation in discriminant function analysis according to a combination of biomarkers from PC1-4 that were pooled for this analysis: Note a separation among all groups, particularly PM0 from the other three groups (PM1, M0 and M1) by a biomarker combination of FSH and leptin (Wilks’s lambda <0.001, in canonical functions 1 and 2). PC: principal component; PM0: premenopausal non-obese; PMI: premenopausal obese; M0: postmenopausal non-obese; M1: postmenopausal obese.

We also evaluated the discriminatory potential of biomarkers displayed in PC1-4 (as shown in Table 3) for distinguishing the subgroups PM0, PM1, M0 and M1; however, no combination of at least two markers reached statistical significance. Thus, subsets of biomarkers from all PCs were pooled for discriminant function analysis, the results of which are illustrated in Fig 3. A combination of FSH and leptin better separated the four subgroups (Wilks’s lambda < 0.001, in canonical functions 1 and 2). FSH was more significantly responsible for separation in function 1 and leptin in function 2.

## 4. Discussion

In the present study, we investigated whether a combination of gonadotropins, sex hormones, serum metabolic risk and inflammatory markers could distinguish obese from non-obese women with different menopause status. It was observed that gonadotropins and sex hormones did not significantly vary among obese and non-obese women and no remarkable correlation was shown between these hormones and metabolic or inflammatory biomarkers. The majority of metabolic risk markers did not vary among the study groups or showed only discrete variations. Inflammatory markers remained unchanged. Using discriminant function analysis, it was possible to identify a combination of FSH and leptin as an independent predictor of obesity in pre-and postmenopausal women.

It is important to note that in both the pre- and postmenopausal group, the medians of BMI were similar and, despite age differences, patients of the two groups were disease-free as clinically confirmed. The postmenopausal status corroborated with higher levels of gonadotropins as it was expected. The increase in FSH and LH releasing that occurs following menopause has been associated with several deleterious biological effects, thereby promoting an increased risk for bone loss, obesity, CVD and cancer [19–21].

Indeed, emerging evidence has suggested that the blockage of FSH activates brown adipose tissue, enhances thermogenesis and reduces adipose tissue in mice [22]. In the current study, no differences in FSH/LH levels were shown between obese and non-obese women, either pre- or postmenopausal, in univariate analysis. However, in correlation analysis, FSH was negatively correlated with BMI, which is a conflicting finding considering that, in a prior population-based study, LH/FSH ratio was not associated with obesity in postmenopausal women [23].

The reduced levels of estradiol and progesterone observed in postmenopausal women were expected. After menopause, estradiol levels remain consistently low and progesterone is no longer produced [1]. Interestingly, obese premenopausal women presented with statically higher levels of estradiol when compared to obese postmenopausal females; however, the relationship between estradiol levels and weight is controversial. It has been observed that premenopausal women with low estrogen levels have lower body fat than postmenopausal women with high estrogen levels [24], and a recent trial demonstrated that weight loss decreased estradiol levels in obese postmenopausal women [25]. Nevertheless, a longitudinal study showed that changes in either BMI or leptin levels did not account for the variability of estradiol in repeated measurements [26].

Regarding the comparison of lipid profiles and fasting glucose among the groups, it was shown that only TC and LDL-c significantly varied between pre- and postmenopausal women. In a recent study carried out at outpatient facilities of the same hospital of the current study [17], postmenopausal women also presented with increased levels of VLDL-c and TC. Similarly to our findings, these authors observed no differences in HDL, fasting glucose levels and hs-CRP. Prior reports from other populations have observed changes in lipid profiles and a high prevalence of metabolic syndrome among postmenopausal women [7]. The large study by Auro et al. [27] found some lipid markers, including VLDL-c and LDL-c, to be predictors of menopause status.

Nonetheless, other authors have reported similar lipid and fasting glucose profiles when comparing pre- and postmenopausal women [28]. It is worth mentioning that we did not observe any significant difference when subgroups (PM0, PM1, M0 and M1) were compared and no lipid marker correlated to the BMI. One thereby can hypothesize that metabolic changes might precede the weight gain that occurs following menopause. Furthermore, it was observed that fasting glucose was positively correlated with the BMI only in the postmenopausal group, which suggests an effect of longer exposure to metabolic risk factors.

The inflammatory biomarkers herein analyzed did not significantly differ between pre-and postmenopausal women (PM versus M) or when subgroups (PM0, PM1, M0 and M1) were taken into account. IL-6 levels correlated to BMI only in postmenopausal women, but both IL-6 and hs-CRP showed no marked interaction in the majority of analyzes. A previous study found a correlation between lipid markers and IL-6 [29], and this might explain the positive correlation observed here. Notably, a high-intensity training intervention was shown to increase IL-6 while reducing visceral adiposity tissue in postmenopausal women [30]. Thus, the roles of obesity, aging and menopause in IL-6 levels need to be further explored.

Finally, we found leptin level to significantly vary between subgroups in univariate analysis. Non-obese premenopausal women (PM0) had lower leptin levels than non-obese postmenopausal participants (M0). This suggests that leptin might be a candidate biomarker of subclinical metabolic dysfunction linked to menopause, considering that it was correlated to BMI in both pre- and postmenopausal women. Moreover, obese postmenopausal women (M1) showed increased leptin levels as compared to non-obese women (PM0 and M0), demonstrating that leptin can also distinguish this subpopulation of females.

Adipokines such as leptin are hormones secreted primarily by white adipose tissue, whose levels have been linked to the amount of body fat [31]. In a prior report, the level of this hormone was positively correlated with BMI, TG and LDL-c and inversely correlated with HDL-c [32]. Interestingly, in this study, we found a correlation between leptin and BMI in both groups; however, in postmenopausal women, leptin was also correlated with both VLDL-c and fasting glucose. Plasma leptin may even be involved in the development of hot flashes experienced by menopausal women, with participation of insulin resistance in this mechanism [33].

Overall, through the discriminant function analysis performed, it was possible to obtain a combination of biomarkers (FSH and leptin) with the greatest discriminant capacity, within the variables tested, for explaining the variance between obese and non-obese women with different menopause status. This could be interpreted as an association between a biomarker that well defines the age-related transition to menopause and whose increasing levels are linked to weight gain (FSH) with a biomarker that has been widely related to obesity and metabolic risk (leptin).

It is worth mentioning that this study has several limitations. First, the cross-sectional design and the fact that repeated measures of biomarkers were not performed preclude us from establishing a direct effect of either FSH or leptin on weight gain among middle-aged women. Sample size, particularly in the groups of obese women (PM1 and M1), was reduced and we thus stress the need for developing population-based cohort studies in this regard. Food habit was not evaluated in the present study, which does not allow us to draw conclusions regarding the role of dietary intake in the relation between hormonal biomarkers, obesity and menopause. This issue deserves to be addressed in future research.

## 5. Conclusion

In summary, a combined analysis of circulating hormonal biomarkers may significantly distinguish obese from non-obese women with distinct menopause status, though correlations with other clinically established markers were not well defined. Hence, a population-based longitudinal assessment is necessary to clarify this potential predictive ability and to investigate novel biomarkers with clinical utility.
